# A Novel Framework and Enhanced QoS Big Data Protocol for Smart City Applications

**DOI:** 10.3390/s18113980

**Published:** 2018-11-15

**Authors:** Shalli Rani, Sajjad Hussain Chauhdary

**Affiliations:** 1 Chitkara University Institute of Engineering and Technology, Chitkara University, Rajpura 140401, Punjab, India; 2 Department of Information and Computing Technology, University of Jeddah, Jeddah 21589, Saudi Arabia; shussain1@uj.edu.sa

**Keywords:** energy efficiency, Big Data analytics, QoS-IoT, Internet of things, Smart City, WSN, green computing

## Abstract

Various heterogeneous devices or objects will be integrated for transparent and seamless communication under the umbrella of Internet of things (IoT). This would facilitate the open access of data for the growth of various digital services. Building a general framework of IoT is a complex task because of the heterogeneity in devices, technologies, platforms and services operating in the same system. In this paper, we mainly focus on the framework for Big Data analytics in Smart City applications, which being a broad category specifies the different domains for each application. IoT is intended to support the vision of Smart City, where advance technologies will be used for communication to improve the quality of life of citizens. A novel approach is proposed in this paper to enhance energy conservation and reduce the delay in Big Data gathering at tiny sensor nodes used in IoT framework. To implement the Smart City scenario in terms of Big Data in IoT, an efficient (optimized in quality of service) wireless sensor network (WSN) is required where communication of nodes is energy efficient. Thus, a new protocol, QoS-IoT(quality of service enabled IoT), is proposed on the top layer of the proposed architecture (the five-layer architecture consists of technology, data source, data management, application and utility programs) which is validated over the traditional protocols.

## 1. Introduction

During the last decade, tremendous efforts have been made to explore Internet of Things (IoT) applications, where communication between heterogeneous physical objects and sensors can inter-operate in integrated environment or platforms. Recent advancements have given a boost to the idea of Smart City. It has been perceived as the intellectual and open environment where citizens can enjoy a quality life, further enhancing research interest in IoT technology and standards and providing a building block to these application scenarios. To facilitate intelligent communication among the different objects of the Smart City, there is growing need for gathering, analyzing, processing and presenting data continuously from all devices with partaking sensing mechanisms.

This bustle requires substantial efforts to tackle the heterogeneity in perspectives to deal with open, dynamic and hazardous deployment conditions. A pertinent objective is to develop a novel, autonomous and compliant model for smart cities, which may interact with diverse domains of the applications such as security surveillance, environmental monitoring, structural monitoring, etc., which could facilitate citizenship and urban living. Powered by the adaptation of various technologies such as sensor nodes (SNs), IoT has stridden out of its beginning phase and is revolutionizing the current frameworks into integrated structures of IoT and wireless sensor networks (WSNs, strong and base layer of IoT framework). The huge amount of data (Big Data) gathered by sensing nodes will be distributed across the varied applications and platforms in the Smart City. There is no formal definition of Smart City, however, we can presume its aim is to develop a city for the efficient use of resources and to enhance the quality of service for the people. This objective can be gained with the help of IoT where WSN plays a crucial role.

A metropolitan IoT will bring a lot of benefits in the administration of public and traditional services such as transportation, parking, lightning, agriculture, safety, etc. The availability of diverse types of data gathered by metropolitan IoT will be exploited to enhance the transparency and local government would be able to take the actions accordingly. Participation of the citizens will be enhanced and will stimulate the invention of novel services. Moreover, in an industrial survey [1], the authors reviewed the literature from the industrial perspectives and mentioned the need of metropolitan IoT for the small to large business organizations which range from start ups to the well-established corporations. The need for the IoT in industrial marketplace, trends and openings are also elaborated.

IoT is transforming heterogeneous objects from being traditionally intelligent by integrating its abundant and prevalent computing, embedded devices (e.g., actuators, smart phones, networked enabled smart devices, etc.), sensor networks, Internet/WSN protocols and applications to transform human life. In the future, it will integrate billions of devices along with objects and embedded systems. Consequently, the Internet of Things (IoT) will considerably increase in scope and size, presenting new challenges as well as opportunities [2].

The IoT is considered the future of the Internet. Consequently, it leads to the rise of concepts such as smart homes, smart transportation, smart agriculture, smart health, smart buildings, etc. converting traditional cities into smart cities where diverse electronic appliances and objects are interconnected with each other to gain high-quality two-way interactive multimedia services. In these cities, where many devices will be connected, a massive quantity of data will be generated, known as Big Data. To enrich smart cities applications, Big Data analytics can play a crucial role in the advancements of information and communication technologies (ICT). The Big Data gathered from various places give a better understanding of the present as well as the future and lead to accurate planning and development. Analysis of Big Data provides an insight into the major perspectives of the smart applications. Smart City applications can consider the integration of sensors and actuators which are auto configurable which can be controlled remotely with the assistance of Internet. Many sensors sense the huge number of data and transmit them to the remote center where they are processed, analyzed and predicted or used to respond user queries. Many studies [3,4,5,6,7,8] have been conducted recently on Big Data. The notion of the Smart City is extendable to the Smart World where the Countries Domain, Cities Domain, and Service Domain are integrated to benefit people all around the world. However, for the Smart City concept, various other concepts such as inter-connected vehicles, GPS, roadside units. etc. are still under development. In the future, around 70% of population will live in cities. With this volume of population billions of devices will be required to communicate with each other and will generate massive amount of Big Data. Therefore, data analysis will be based on the user requirements and priorities which will make cities smarter. Hence, diverse technologies used in applications and their data analytics are driving the IoT out of its initial stages and revolutionizing it, therefore IoT is migrating from traditional framework to fully integrated novel future network.

The WSN and its various technologies are soundly integrated into a metropolitan infrastructure, forming a digital skin [9]. The huge amount of information generated by the prevalent embedded devices will be shared across the different platforms and applications to convert the cities into smart cities and forecast the development. Typically, to achieve the urbanization, it is important to realize the requirement of service outlining to improve the efficiency and city management schemes. Recently, many organizations are in the queue for setting up platforms for live monitoring, scheduling and collecting urban process attributes. All these activities are accomplished by gathering data in two ways; offline and real-time (for Big Data processing, analysis and decision making). Data gathering techniques are costly and complex. Consequently, there is a requirement to include intelligent technology which can proficiently and speedily gather many data, accomplish analyses on Big Data, and envisage the future to enable improved planning and development [10,11]. Considering the above-mentioned facts about IoT and the role of Big Data in Smart City applications, in this paper, we propose a Smart City Big Data protocol for M-IoT where WSN plays a fundamental role in gathering the huge number of data. A complete architecture to develop the Smart City applications is presented which is based on IoT based Big Data analytics.

This protocol is based on data that will be managed and gathered by the sensor nodes in WSN. However, WSN is constrained by many issues, which should be resolved for the real time communication in M-IoT. Most of the applications (environment monitoring, border security surveillance, transportation, etc.) in M-IoT require energy efficient and real time communication. To overcome the various challenges faced by Big Data, much research has already been done but not from the perspective of scalability (IoT applications will cover a huge area of city). Traditional protocols developed for WSN will not provide a strong platform to M-IoT; therefore a novel approach is required to cater present need. In previous work on WSN [12], areas are monitored on the basis of queries. Queries-based applications are the most prominent part of the M-IoT. However, queries (users queries from the data server, i.e. Big Data) are not handled in the accurate way and it is not suitable for real world scenarios for two reasons. (1) They do not consider the priority of the query messages. Consequently, query responses will be dropped along with query requests.Thus, the whole system will malfunction. Most of the work on smart cities is based on Big Data only. Smart City citizens require access to real time Big Data, hence loss of messages and delay in response will not be tolerable due to WSN constraints. (2) The header nodes, which are responsible for the forwarding of data, are dynamic. Furthermore, all nodes have different levels of energy. However, static and homogeneous nodes can simplify the processes in real world applications.

In this paper, we propose a framework for Smart City Big Data analytics and an enhanced quality of service based protocol (QoS-IoT) for IoT applications.A five-layer architecture is presented, which is capable of analyzing the huge IoT dataset. Moreover, the timely delivery of data makes the decision making process efficient. The proposed system is evaluated in terms of throughput, energy and transmission time.

The rest of the paper is organized as follows. In Section 2, background and motivation is discussed. In Section 3, we present the technologies used in Big Data. Framework for IoT based Smart City with Big Data management is provided in Section 4 followed by the proposed model in Section 5. In Section 6, concluding observation is presented.

## 2. Background and Motivation

Due to digitization, cities are converting into smart cities, where cities are equipped with various electronic equipment used by many applications such as street surveillance cameras, transportation monitoring, intelligent health system, etc. Some applications already use established systems to handle the smart concepts, such as Global Positioning System (GPS). It adds on the power to the handheld devices to generate a scenario where data are exchanged based on locations. Therefore, in this type of environment, queries based on features, objectives, security, etc. required to be resolved [13]. These can be outlined as below:

(1) Reliability: How to handle the uncertainty due to real time constraints and offline dynamics and how the quality of the data can be ensured.
(2) Intelligent Transmission: How objects can become intelligent to transmit the data and design of the new objects.
(3) Less Delay: How the response time can be reduced.
(4) Data Gathering: How data should be gathered in the lowest possible time and at low cost (in terms of energy, fault tolerance, throughput, standard deviation, etc.) and how this objective can be obtained when many sensors are deployed around the environment.
(5) Processing of Big Data: Which schemes can be implied for the Big Data processing and analytics (online data analytics (OLAP) and online data transaction processing (OLTP) for real time communication.

Based on these considerations, Smart City applications exploit the capabilities of ICT which help the citizens in the efficient utilization of limited resources. Various organizations are using advanced systems and the majority of these technologies are comprised of efficient storage capabilities and sensing schemes for exceptional quantity of data. The fundamental objective behind the present approach is the vast deployment of sensor nodes to gather the data in an efficient way. The design of this system needs all the features of WSN to analyze, gather and transmit the data of all the objects. Integration of many data poses many constraints and efficient techniques of Big Data analysis are already developed. However, for large-scale applications, in some circumstances (posed by environment), a major portion of the data can be disjointed, which can lead to unreliability and requires better urban planning and a novel scheme to avoid it. Smart M-IoT framework can provide a new way to integrate the resources on the basis of geographical locations and can then be analyzed by novel system to provide diverse services to the citizens. Citizens can be benefited in terms of health, security, mobility, pollution, etc. Many projects are available in the literature, such smart car parking, dynamic lightning system, car locking system, etc., where sensors play a major role (specific to the applications). Other service oriented applications for Smart City applications in IoT are vehicle to vehicle communication, air/noise pollution, health care systems, real time driver assistance, potholes monitoring, etc. Recent research is very constrained due to simple design issues of IoT. No single system is fully developed and authorized to support scalability and efficient communication (Big Data transmission and gathering). To derive knowledge about the various aspects of Smart City, Big Data analytics of old as well as new information is required. Therefore, the role of WSN in Big Data analytics cannot be ignored.

WSN provides all the necessary data to the infrastructure of IoT, which are used in all Smart City applications. This integration is particularized in [14]; however, the focus of the paper is on network only. Data, which have the most crucial role and around which the whole IoT system revolves, are not considered. The context and instructions of communication for data are emphasized by the authors of [15]. They outlined the necessity of semantic gloss of IoT, which motivated us to propose a model approach where sensor nodes can slog with IoT framework and attention is given to the data. Overlays are formed over WSNs in [16], for the upliftment of urban data in IoT and proposed a solution for the delivery of urgent data. However, they did not consider the auto response from the nodes, which can further reduce the delay in the process. A framework for urban system is proposed in [13] based on the support of sensors and network, using data and cloud integration of heterogeneous devices. It focuses on the noise based Smart City application and can be applied to other services. A survey on the protocols, standards, and architecture for urban IoT has been made in [17]. It presents the technical solutions and guidelines for the Padova Smart City project. Looking into the problem of manhole cover, H.H. Aly et al. presented the solution with an automated monitoring system which is a fragment of Smart City and IoT [18]. Underground security issues for manhole cover are covered by automated and non-automated systems. A distributed sensing algorithm, which deploys the sensor network on top of IoT devices, is proposed in [19], which is based on sensor parameters. Event driven architecture for IoT domain was proposed by authors [20]. Different approaches of event triggered methodologies are also conferred to address the efficient phases of event driven data in IoT. In this paper, we presented a framework for the continuous monitoring of Smart City with reliable and fast communication system, which will function on IoT in an energy efficient way (essential requirement of Big Data). The comparison with energy efficient algorithm for IoT (ME-CBCCP [21]) and other traditional algorithms, as presented in [22], provides validated insight into the proposed scheme.

## 3. Challenges and Management of Big Data Using Emerging Technologies

In the processing, analyzing, filtering, etc. of gathered data, management is a vital process. A problem was first raised a long time ago during the efforts of UK e-scientists: data were distributed over geographical regions and owned by various entities [4]. To handle this problem, scientific data life cycle management (SDLM) model was proposed. This model analyzes the already existing approaches from different perspectives. The traditional model follows the same steps to handle the data, planning, data gathering, filtering, processing, feedback and documentation [58,59,60]. The following is a discussion of the general stages of handling Big Data.

### 3.1. Raw Data

Agencies, data server centers, researchers and organizations incorporate the gathered raw data and enhance the value of data by using inputs of individual developed programs and novel reach projects. The data are first converted and then stored in the form of value added services. No standard has been globally accepted to store and administer the data. The data are generated with specific attributes which depend on the programming done to handle the data.

### 3.2. Data Processing

Sensor nodes deployed in WSN gather data, which is the very first stage of the Big Data life cycle. In IoT, data are also gathered from mobile phones, satellites, laboratories, blog messages, etc. Specific techniques are implemented to gather the raw data from the integrated environment of IoT. Data can be processed before transmission (sensor nodes) or at data center (data server or base station (BS)). Transmission of processed data consumes less energy and time, which are the foremost constraints of WSN. Data generation in IoT depends on the requirements and life of the citizens. Data in IoT are also based on expressions, habits, and emotions, which are accumulated with the help of Internet, and sensing technology again plays an important role at this place. This type of data is required in health departments. Scientific Data Infrastructure (SDI) organization must [58,59] consider the problem of heterogeneous data. Generally, the following methods are used to gather data [23]:

(i) Sensing: Sensors are deployed randomly or manually at fixed locations to gather the data to measure the physical attributes, and converted into the digital signals for transmission and storage. This data may be of any type, e.g. temperature, humidity, pollution, chemical, medical, security, etc. Sensed data are transmitted to the BS with the help of wired or wireless networks. For Smart City projects, both networks are required. The wired network is the network of base stations or the data centers where the gathered data (video surveillance, patient health monitoring, etc.) are processed. The data are gathered with the help of wireless networks from remote areas. That is why WSN is known as the backbone of IoT. WSN has gained a lot of attention and has been used in many fields such as underwater monitoring, civil engineering, wildlife, health care, surveillance, etc. Various techniques are developed [12,14,15,16] to utilize the capabilities of WSN in efficient ways.
(ii) Data Capturing Techniques: Data in IoT are captured by integration of task, word segmentation, and index and web crawler. In search engines, web crawler stores/downloads the pages, and linked information is accessed through the uniform resource locater (URL). Data from various applications are accessed by caching and search engine optimization techniques. Many techniques have been proposed recently for efficient extraction strategies (for high quality data) to address numerous Internet applications.
(iii) Log Files: Data are collected automatically by recording the manipulation steps and processing via data source management system. Log files are maintained by all companies (IBM, Infosys, TCS, etc.) where work is carried out on digital data. The advantage of log files is in backing up the system, in case of any failure. Timestamps are used to roll back the crucial transactions. Data are also recorded on the web by user’s visits on the web pages, clicks on links, etc. Data are all in the ASCII format to enhance the query efficiency in huge data warehouses.
(iv) Zero-Copy (ZC) Packets: Data copies exchanged between the nodes are not copied due to this technology. Data are generated from user nodes and routed through the network interfaces and sent to the BS. Data are transmitted to the user nodes which are accessed from the BS. Data are not copied in between the system calls. Direct memory access (DMA) is used to avoid data copies and reduces the number of system calls, hence time.
(v) Smart Phones: Mobile phones have been replaced by smart phones, which are gradually becoming more powerful. Data acquisition techniques are enhanced and various different parameters are produced. These devices are capable of gathering information about weather, health conditions, location, and can capture multimedia data. Mobile Internet technology is becoming popular among the people due to its capability of gathering and transmitting Big Data in smarter way.

Along with various methods mentioned above, other technologies such as cloud computing are also assisting in handling the data processing.

### 3.3. Analysis of Data

This process empowers any organization to gather plentiful information, which can affect the various processes of business. This process is very complex due to heterogeneity of data and scalability of algorithms. It helps them in understanding the relationships among the data and their features. It enables developing new methods of data mining to predict the future. Over time, new techniques have facilitated speedy accessing and mining of two types of data: structured and unstructured. Analytical techniques can be categorized into data mining, statistical analysis, visualization and machine learning. Data mining is useful in many application of medical and engineering. Big Data involves different data formats, and data should be accessed in minimum possible time. An efficient architecture should support all data analysis techniques and data formats. High performance algorithms and protocols which are developed for WSN or IoT can help achieve these objectives. Many traditional data analysis techniques can be applied on Big Data: cluster analysis, data mining techniques, correlation analysis, statistical analysis and regression analysis.

With the increase in the size of data, new methods should be developed to gather, store, analyze and process Big data efficiently. The various challenges in Big Data analytics are described below:

1. Heterogeneity of Data Formats: Data mining algorithms search the unknown patterns and various homogeneous formats for the analysis in structured way. However, analysis of semi/unstructured formats is complicated. Data should be structured before the analysis process. The variety of data is the big issue in Big Data. Information of data may not be structured and may not be well organized (relational database), as data are collected from the various sources.
2. Correctness: Data gathered from the small sets of applications can be seen or perceived as accurate data. However, Big Data collected from the various resources cannot be considered as accurate as the volume adversely affects them.
3. Scalability: The huge number of data per se is an issue for Big Data analysis. This issue can be mitigated with the help of processing speed. However, the number of data increases more quickly than CPU and resource speed. Many computing resources are shared by the nodes along with memory and processor. Scalable systems are required to deal with these kinds of issues.
4. Data Complexity: Different types of data, such as structured, unstructured and semi-structured data, pose another challenge to Big Data analysis. Structured data have similar format, predefined range/domain and processes. Data are generated with the help of sensors or computers without human intervention. Data are processed with database query languages such as SQL. However, Big Data gathered from the multimedia resources are unstructured and software such as Hadoop can handle this type of data. Hadoop analyzes and clusters unstructured and semi-structured data with the help of MapReduce. Extraction of important data is a serious challenge. It is difficult to validate all the data items in Big data because data sources are varied temporally as well as spatially in gathering and format. The metadata description of data cannot be controlled and may or may not be accurate. It requires inspection and critical analysis of data.

### 3.4. Publishing and Sharing of Data

To benefit the public, governments, researchers, agencies, etc., data and their resources are gathered, analyzed and published. Large and wide datasets of Big data must be stored with easy accessibility, reliability and availability. Storage space should be managed to apply DBMS techniques efficiently. Stored data should be consistent (with the help of data center), and easily available. Data should not be lost due to network obsoleting (main constraint of WSN).

### 3.5. Privacy and Security

Citizens of Smart City require that their data on cloud be safe and private. Intellectual property rights should be developed to maintain security, privacy and confidentiality of data, e.g., hospital patient data are confidential and, if Big Data techniques and IoT protocols are involved in the patient data monitoring, then some security techniques are required to manage their digital data. Data integrity is also an important factor for joint analysis where data analysts and decision makers share information. Data mining techniques are required to improve decision making processes and cooperative tasks on Big Data.

### 3.6. Discovery and Re-usability of Data

Data access schemes are required to ensure the quality, validation, value addition, and data conservation by reuse of existing data and discovery of new data. It involves many fields within it, such as archiving, representation and data management. Schema and relational model are used for structured data management and to improve reusability of data.

The above sections discuss the various techniques and issues involved in Big Data; some of the issues can be solved by the use of novel methods and techniques. In the next section, we present the proposed framework to handle the data in energy efficient (in the venergy constrained environment of WSN) ways for Smart City applications.

## 4. A Framework for Big Data Analytics in Smart Cities Applications of Iot

IoT based Smart City should facilitate fast access to data along with accurate information. For the Smart City development, several sensors are required to gather the information along with other IoT devices. To connect the Smart City devices and IoT system, other devices are required such as edge nodes, aggregators, data server, etc. Sensors generate the information at high speed and Hadoop system is required for the same. As per the requirement and constraints of WSN and IoT, we have deployed the QoS novel protocol to gather the data efficiently (Big Data). Architecture of the proposed system is discussed below to show the efficiency of the system.

### 4.1. IoT Based Smart City

**Figure 1:**
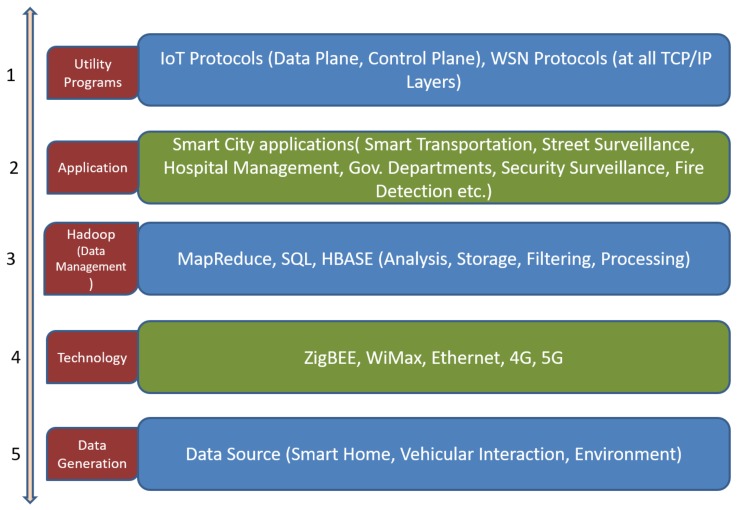
Structure of Smart City Big Data Analytics.

The main challenge in front of Smart City projects is how to use the IoT system in these projects. IoT is connecting various heterogeneous objects and data come from the many heterogeneous resources. In the digital era, all devices are required to connect with high speed Internet and data from sensors should come with in the shortest possible time. The main aim of deployment of sensor nodes is to gather information, even from remote areas. Smart parking, pollution measurements, detection of unsafe events, smart driving, smart homes, smart streets, smart health monitoring, etc. are some of the applications that are useful for citizens of smart cities. Sensors play a major role in implementing these applications. Sensors are constrained by many features such as energy, scalability, processing and low memory. Some techniques are required to overcome these constraints, as in IoT system these constraints pose major challenges. Data for all the applications are required for real time analysis. However, for real time data and processing, applications should gather the data in minimum possible time and the data should be reliable. These applications not only require the location information (GPS), but also require other information such as temperature, humidity, patient symptoms, security intruders, etc. The proposed approach gathers the information in energy efficient way, without network obsoleting. The architecture of the Smart City is shown in Figure 1, where utility programs are installed on the topmost layer of framework. These protocols exploit the benefits of IoT by optimizing the existing techniques of data gathering, transmission, processing and analysis. These protocols are required for the accurate working of the applications by reducing the standard deviation and enhancing the reliability. Hadoop and Mapreduce are required for Big Data analytics along with supporting technologies. Data sources are the base of this framework where sensors are deployed to generate the data.

### 4.2. Smart City Planning and Implementation

**Figure 2:**
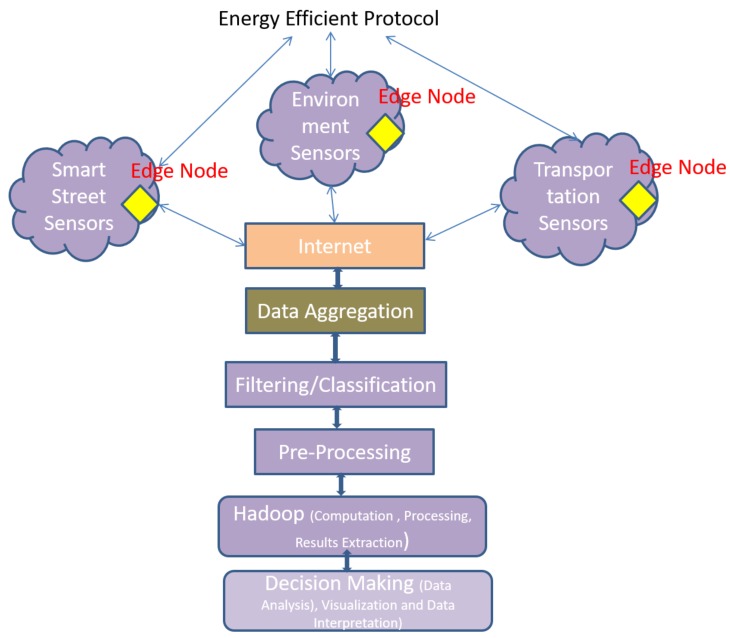
Implementation framework.

For planning and implementation of the novel framework, we have considered the layers proposed in the Figure 1, with one exception: data are generated by sensor nodes (not from log files, auto systems, etc., as mentioned in Section 3). To propose the optimized protocol as a utility for IoT Smart City projects, a novel QoS based protocol is developed for the sensor nodes for the cooperative data transmission, for energy efficient communication (green computing in IoT). This approach is necessary to fulfill the demands of the citizens, e.g., to build the smart streets and smart homes scenarios, governments analyze the needs of people by analyzing the energy consumption from historical data and can plan for better services in the future. In another application, for the healthy environment, information about pollutants is required which should be available in real time, which is possible only when sensors are deployed randomly, and data are gathered and transmitted without human intervention in optimal way. In dynamic traffic light system, traffic is controlled by real time data, where reliability of the information is crucial. All these constraints are related to the sensor nodes and, in another way, to the IoT, as WSN is the foundation of IoT system. In IoT, abundant number of sensors is required to control the information and this information acts as the part and parcel for Smart City scenarios. Therefore, in this paper, we have proposed a novel approach to transmit the data in efficient way to optimize the energy parameter, as shown in Figure 2.

### 4.3. Big Data Analytical Architecture

The data are gathered by the sensor nodes from data sources where they are deployed. This module gathers and transmits the data to aggregator system (BS) via Internet. Citizens do not have or have limited access to the results of data. A five-layer system is involved in the Big Data processing, as shown in Figure 1. The bottom layer is the data source layer, which gathers the data from the Smart City resources. This layer is based on the Hadoop system. The data storage is done on the Hadoop system by using MapReduce. The data are transmitted to the third layer by using ZigBee, WiMax, 4G or 5G. The data are managed by the third layer which is used by the various smart applications at the top. However, data gathering and transmission require the inclusion of WSN/IoT protocols, which are in the first layer. These protocols act as the utility software because they reduce the standard deviation, and enhance the energy management, network lifetime, scalability and reliability. Afterwards, decision making process is executed on the data that are collected at the data center /aggregator (BS) after the process of data filtering, as shown in Figure 2. The five-layer structure is explained in detail below:

1. Data Source: This layer handles the data, generated by various IoT sources such as sensors and objects. Data are gathered at the sensor nodes and these data are processed before data transmission to remove the errors (if any) before data transmission. Data compression techniques can also be applied on the sensor nodes to reduce the required bandwidth for the data and to enhance the data transmission from other nodes (reduce network congestion). The data produced in this layer are heterogeneous. Along with all other techniques, security can also be maintained in this layer by encryption/decryption techniques. Data filtration removes the unnecessary and redundant data.
2. Technology: This layer is responsible for communication among the sensor nodes, the edge nodes and BS, and it relies on the ZigBee, WiMax, etc. In IoT there are several BSs connected with each other through the Internet.
3. Data Management: This layer is required for the data analytics and is responsible for data management. Third party tool is required to integrate Hadoop within the system for implementing the model. All gathered data must be stored in Hadoop using MapReduce. Analysis of data is performed in this layer.
4. Application layer: The analytical data and reports generated at the third layer are used by the application layer by end users.
5. Utility Programs: WSN and IoT protocols are used at the topmost layer for the efficient working of other layers (data gathering, transmission, encryption, processing, etc.) Traditional protocols do not suit the scalable network of IoT. New techniques are required for the efficient working of the architectures. As shown in Figure 2, the protocol is implemented for the efficient working of the sensors. Data are collected by the sensor nodes and transmitted to the edge nodes, which further transmit the data to the BS. Data from all the network areas and subareas are crucial for excellent and accurate decision making. This layer requires fault tolerance and energy efficient communication (to conserve energy for long network lifetime). The proposed protocol is implemented in MATLAB and it is validated over BDEG [22] protocol along with other traditional protocols. As IoT deals with many Big Data, we require a system that will proficiently process and execute large collections of huge datasets. To fulfill these necessities, the Hadoop system can be used, which contains various master nodes along with other nodes under it. In Hadoop, data are divided into equal portions and stored on data nodes. Parallel processing is performed on these nodes by MapReduce. All reports are generated at the Hadoop and decision is taken on these results.

## 5. Network Model and Results Discussions of Proposed Protocol

In this section, the proposed protocol model for smart applications is discussed. The network model and the radio energy model are two standard models that are used in the data processing in any Smart City application.

### 5.1. Network Model

It is assumed that sensor node deployment is randomly uniform in a square shaped area (Table 1). The following assumptions are made for all nodes in the network:

(i) All nodes are considered to be static, which means the nodes do not move once they are deployed. The main objective of sensor network is that nodes collect data from the environment periodically and send to the base station. (ii) All node links are symmetric and begin with the same initial energy. (iii) Each node can merge redundant data. All sensor nodes are assumed to have limited batteries and recharging them is infeasible. (iv) Nodes do not possess any GPS equipment and their relative distances are calculated based on received signal strength.

### 5.2. Energy Consumption Model

The radio model is used for reception and transmission of a l-bit message. Communication energy is consumed at much lower level as compared to the energy consumed on computing and storage process. Energy consumption on communication is considered for simplicity by using Equations (1) and (2) [21].

(1) $$Etx\left(l,d\right)=lEelec+lEefsd^{2}Ford<d_{o}$$

(2) $$Etx\left(l,d\right)=lEelec+lEefsd^{4}Ford>d_{o}$$

For reception of messages, the radio expands (Equation (3)):

(3) Erx(l)=lEelec

To merge the *m* number of messages, the energy consumption is computed as (Equation (4)):

(4) Edx(l)=mlEda

In Equations (1)–(3), Eelec represents the energy consumption of transmit or receive 1-bit message. In Equation (4), Eda represents the energy consumption of merge 1 bit message.

The following threshold values are used: when the distance is less than $d_{0}$, the free space channel model is used ($d^{2}$ power loss); and when the distance is more than $d_{0}$, the multi-path fading channel model ($d^{4}$ power loss) is used.

### 5.3. Results Discussion

**Figure 3:**
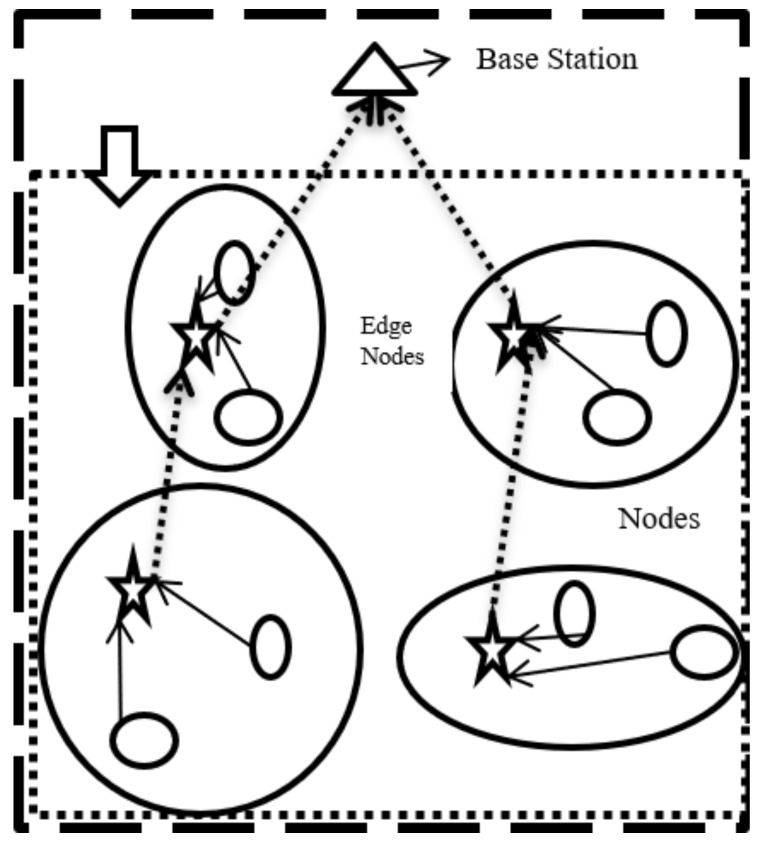
Network scenario.

**Figure 4:**
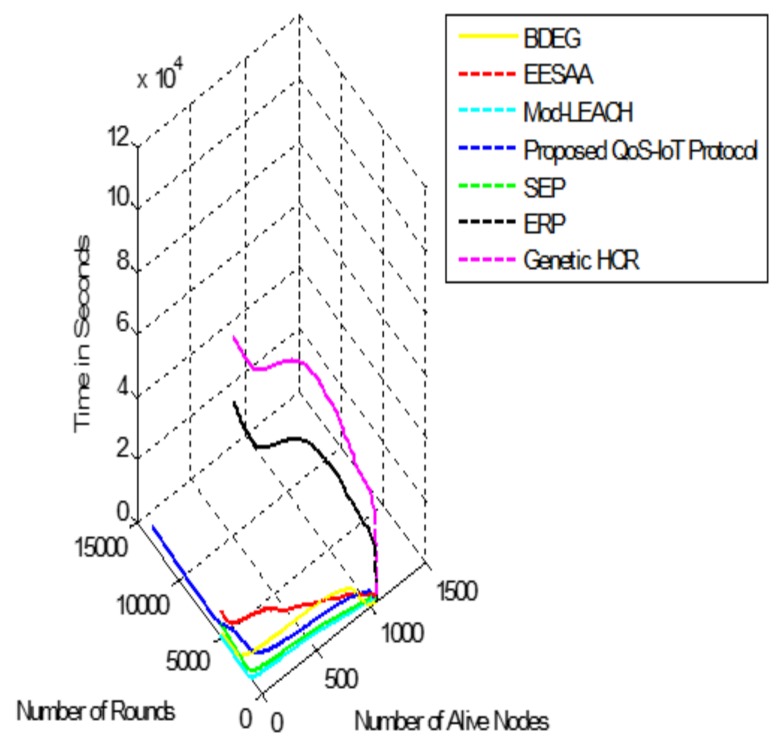
Network lifetime in reference to the remaining energy of nodes.

We have created a network scenario (Figure 3) to gather data from sensor nodes at the BS. The data are transmitted through a predefined algorithm which divides the area into four equal subareas. Data are transmitted to the BS with the help of edge nodes, which are placed in each subarea. Edge nodes help in maintenance of security while transmitting data to the BS/aggregator through Internet. Figure 4 shows the network lifetime of the sensors with remaining energy after transmission of data. The proposed protocol (QoS-IoT) presents better performance than BDEG (Big Data Protocol) and other traditional protocols. The novel protocol still has seven living nodes, as compared to BDEG where only three nodes are alive.

**Figure 5:**
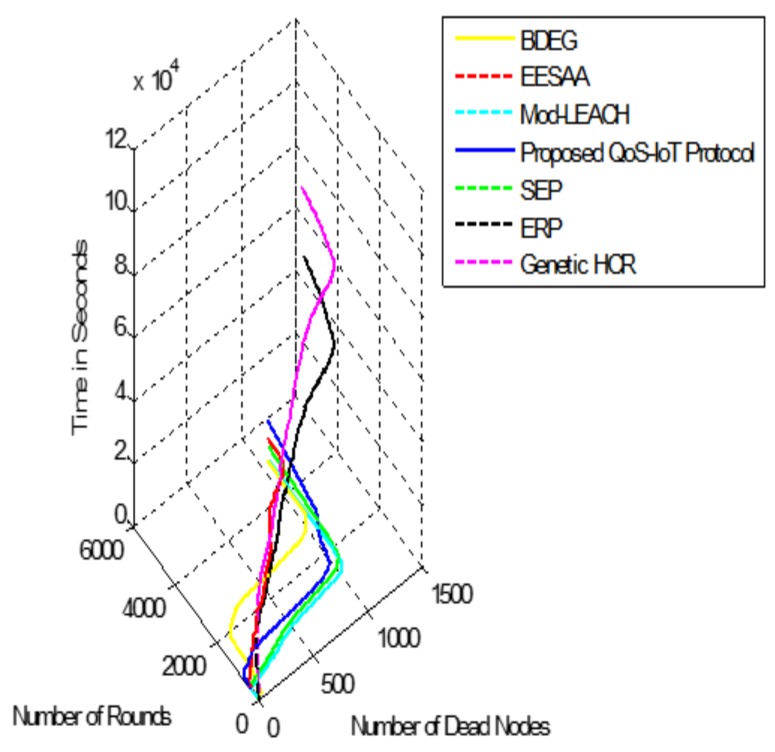
Network lifetime in reference to the energy depletion of nodes.

**Figure 6:**
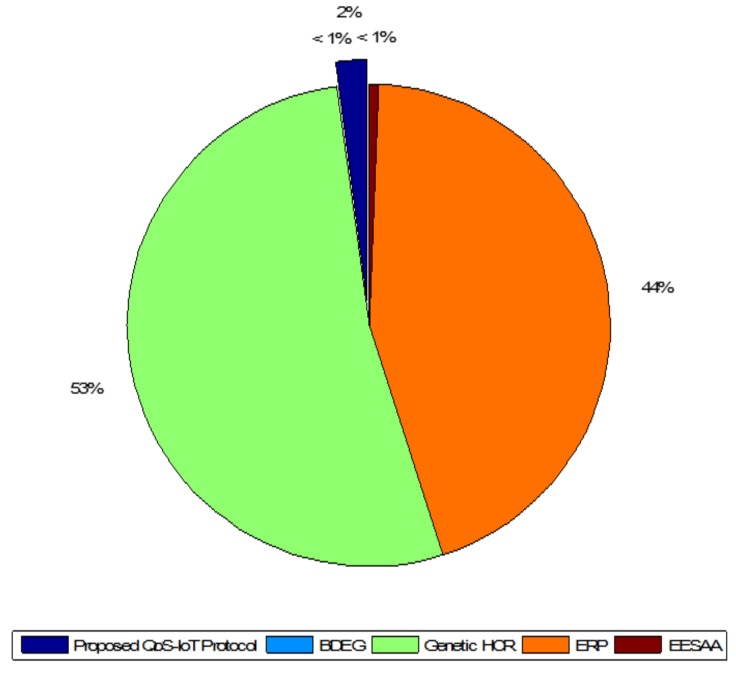
Comparison of variance of the protocols in network throughput.

**Figure 7:**
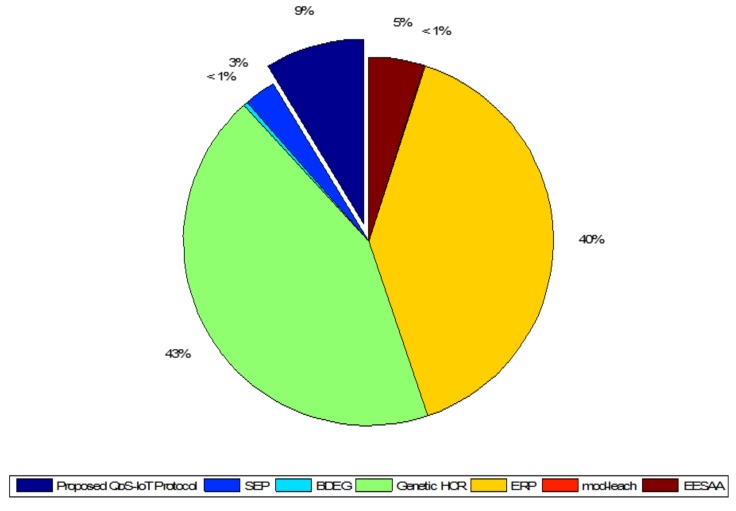
Comparison of standard deviation of the network throughput.

To make this clearer, another comparison of the network lifetime is shown in Figure 5, with dead nodes. Figure 6 and Figure 7 depict the standard deviation and variance in network throughput (number of transmitted packets in one time).

Variance and standard deviations are used to show the distribution of data over population. The variance gives the results in square while standard deviation simply squares the variance. Variance of the network throughput of the protocols can be observed in Figure 6; the higher variance shows the throughput away from the mean. However, as it gives the results in squares, it is not a very useful measure of the transmitted packets; e.g., in Figure 6, the variance of QoS-IoT protocols is 2% while the variances of BDEG and EESAA [21] are less than 1%. It can be acceptable in some situations; however, variance is not a true measure, which is why standard deviation is preferred.

High standard deviation means values are far away from the mean. In network throughput, we require low value of standard deviation because we want packets to be transmitted in uniform manner to efficiently utilize the bandwidth and to prolong the network operation. If its value is high, it shows some of the nodes in the network will deplete their energy long before the other nodes, which can make network obsolete (network partitioning).

Figure 7 shows the standard deviation of the comparative protocols. According to network lifetime, the proposed protocol is validated over the other protocols; however, in network throughput, BDEG and EESAA have better performance because the sensors in two subareas of the network are located far away from the BS and, if the BS is in center, then it will show improvement over both protocols. Figure 7 depicts the true variation of the protocols where QoS-IoT protocolś value is 9%. Considering the long network lifetime and energy efficiency, the proposed approach is better than the other protocols. We consider the cross-layer approach to reduce the standard deviation, as it is the stringent requirement of the IoT.

## 6. Concluding Observation

Smart City applications have a major impact on the development of a nation. These applications will enhance the decision making power of the citizens. Big Data analytics will facilitate intelligent and effective decision making policies. In this paper, we propose a framework for Smart City applications using Big Data generated from the IoT system. The proposed system is composed of a five-layer model, where data are aggregated, transmitted, analyzed, filtered and processed. The major role in this framework is played by the WSN. Sensor nodes generate and transmit the data to the BS. However, they are constrained by many features. To achieve green computing in IoT, it is necessary to conserve energy by using new methods or protocols. That is why, along with the proposed framework, a new protocol, QoS-IoT, was developed to save maximum energy of the nodes for long network lifetime of WSN. QoS-IoT is validated in terms of time, throughput and energy. Throughput of the network is enhanced but with greater variance over time. To reduce the variation of the throughput, a cross layer model will be considered in future work.

## Figures and Tables

**Table 1:** Parameters used in QoS-IoT.

Parameter	Value
**Network coverage**	(200, 200) m
**BS location**	(100, 200) m
**Node Number**	1055
**Initial energy (Quantity) of Normal nodes**	0.5 joules
**Eelec**	50 nJ/bit
**Efs**	10 pJ/bit/m^2^
**Emp**	0.0013 pJ/bit/m^4^
$d_{0}$	87 m
**Eda**	5 nJ/bit/signal
**Data packet size**	4000 bits
